# Supplementary material for: Genome-wide characterization and expression analysis of aquaporins in salt cress (Eutrema salsugineum)
Source: PeerJ. 2019 Sep 12;7:e7664. doi: 10.7717/peerj.7664 (PMC6745184; doi:10.7717/peerj.7664)
Supplement: Table S1 [file peerj-07-7664-s001.docx]

**Table S1** Primers of EsAQPs used in qRT-PCR.

| EsAQP | Primer | Sequence 5′~3′ |
| --- | --- | --- |
| EsPIP1;1 | Forward | CCAGCCTAAGCAATATCAG |
|  | Reverse | GTGGTCGTCCCAGGAATG |
| EsPIP1;2 | Forward | ACCAAAGCAATACCAAGC |
|  | Reverse | ATGGTCGTCCCAAGCGTT |
| EsPIP1;3 | Forward | CTACACTAAGGGCTCC |
|  | Reverse | TGATCGGAATCGTCGC |
| EsPIP1;4 | Forward | CTACACCAAGGGTTC |
|  | Reverse | TATCGGAATTGTCGCC |
| EsPIP1;5 | Forward | CAATCAGGACCGTACCAG |
|  | Reverse | CAGTGATTGGAATAGTGGCC |
| EsPIP2;1 | Forward | GTGTCGTTAGTGAGAGCCAT |
|  | Reverse | CATGGGAATCTCTGGCGCT |
| EsPIP2;2 | Forward | GAGGGATACAGCACAGGG |
|  | Reverse | GGACTCGTTATAGATAACG |
| EsPIP2;3 | Forward | GATACAACACAGGCACTGGC |
|  | Reverse | TAGTGGCCAAGTGTACCATG |
| EsPIP2;4 | Forward | CAGTGCCTTGGTGCTATCTG |
|  | Reverse | GGTGGCTAAGTGAACCATGAAG |
| EsPIP2;5 | Forward | GTACATGGTGGCTCAATGCCT |
|  | Reverse | CCGTGATTGGGATTGTAGCT |
| EsPIP2;6 | Forward | CACTTGCGGAGTTGGTTTGG |
|  | Reverse | GGTTAATTCCGGTGCCAGT |
| EsPIP2;7 | Forward | GGTGAGAGCTGTTGGTTACATG |
|  | Reverse | GAAACCAATTGGAAGTGGAGCC |
| EsTIP1;1 | Forward | TACTGGATTGCTCAGTTAGCC |
|  | Reverse | CGGAGCAATTGTTCCAAGACTA |
| EsTIP1;2 | Forward | ACTGGATCGCTCAGCTTCTT |
|  | Reverse | TGGTGCGATTGTTCCGAGACTT |
| EsTIP1;3 | Forward | CGCTTTCCTATGGAGTCACG |
|  | Reverse | CGGCAATGGCTGCACCAAT |
| EsTIP2;1 | Forward | TTGGTCTTGCTCTCGGTGGTC |
|  | Reverse | GAACCATTCTTGGGATCAGCG |
| EsTIP2;2 | Forward | CATCACCGGTTTCTTCTAC |
|  | Reverse | GACCAGAGCAAAAGTCACCACA |
| EsTIP2;3 | Forward | GCTCCATCGTTGCTTGCCTT |
|  | Reverse | CACCACTGAATGGACCAGCA |
| EsTIP2;4 | Forward | TCAGCTTCTCGGCTCCACCGTA |
|  | Reverse | TCAACGGCTGTGGCATACACT |
| EsTIP3;1 | Forward | GAGGCTCTCAACAAACGGCTT |
|  | Reverse | CCACTAACGTGTTTGCTCCAAC |
| EsTIP3;2 | Forward | TGAGGCTTGCAACTAATGGCTC |
|  | Reverse | GATGGCTAGCGGTGCTATGATC |
| EsTIP4;1 | Forward | TCAGTTGTTAGCCTCATCCGC |
|  | Reverse | GAAGTGGGCCTAACCCATCA |
| EsTIP5;1 | Forward | CAGTGTACACAAGCACGAACGC |
|  | Reverse | TGGACTTCACAGATGCTTGCC |
| EsNIP1;2 | Forward | TGTGTTCGTCGGAACATTACCG |
|  | Reverse | GTTGATCCTACTGCTATTCCGGC |
| EsNIP2;1 | Forward | CGGCCTACTTGACCGTTCAAG |
|  | Reverse | TGACGGTTGCGCCGATGATTA |
| EsNIP3;1 | Forward | GTTATAGCCGCTGTTGCTACCG |
|  | Reverse | TCCATGCACCTGATAATGCTCC |
| EsNIP4;1 | Forward | TTAACCCTGCGGTTACCATCAC |
|  | Reverse | CCGGCTACGAAGACGTTTAAC |
| EsNIP4;2 | Forward | CTCCAATTGATTCCGCTGCACG |
|  | Reverse | GGCCATAACTCCGAGTATAGGAC |
| EsNIP4;3 | Forward | CTCCGGCTGATTCGTCAGCAC |
|  | Reverse | CCAGCCACGAAGACATTCAACA |
| EsNIP5;1 | Forward | CTTCGTTGTTACTGCCGTTGCC |
|  | Reverse | TATGGCACCAAGTGTAGGAGCC |
| EsNIP6;1 | Forward | CCTTATGTTCGTTGTCACTGCCG |
|  | Reverse | AATCGTTAGCTGCAATGGCTGG |
| EsNIP7;1 | Forward | GCGTTGCATTGTGGTCCTCAT |
|  | Reverse | CCGGTCCTAGTGATCGAGCT |
| EsSIP1;1 | Forward | TCAGGTTGATGTGCACACCG |
|  | Reverse | TGTGAGAGCTGTTCATGTATGCC |
| EsSIP1;2 | Forward | CCGTCTCGGTGTTTATGGTTGG |
|  | Reverse | CGGTGAAGGAGCTGATCCAATAC |
| EsSIP2;1 | Forward | TCTGATCTAACCGGCGGATGC |
|  | Reverse | CCAAACAGCGAGCAGTGTAGC |
| Actin | Forward | GCACAATCCAAAAGAGGTATTCTCACCT |
|  | Reverse | GGAGCCTCGGTAAGAAGAACAGGG |
