# Supplementary material for: Genome-wide characterization and expression analysis of aquaporins in salt cress (Eutrema salsugineum)
Source: PeerJ. 2019 Sep 12;7:e7664. doi: 10.7717/peerj.7664 (PMC6745184; doi:10.7717/peerj.7664)
Supplement: Table S2 [file peerj-07-7664-s002.docx]

**Table S2** Primers used in construction of vectors. The restriction sites were drawn with horizontal lines.

| vector | Primer | Sequence 5′~3′ |
| --- | --- | --- |
| PBI121-EsPIP1;2 | Forward | CCTCTAGAGAGAGAGACACA |
|  | Reverse | GTGTCGACGCTTCTGGACTTGAATG |
| PBI121-EsPIP2;1 | Forward | GCCTCTAGAGCACATGTGCTTCTC |
|  | Reverse | TAGTCGACGACGTTGGCGGCACTTC |
| PCS107-EsPIP1;2 | Forward | TAGGATCCGGAGATGGAAGGCAAG |
|  | Reverse | CCGGAATTCAATCAGCTTCTG |
| PCS107-EsPIP2;1 | Forward | CTGGATCCACACAACAGAGAAGC |
|  | Reverse | CCGGAATTCAGGATCTGCTTTAAT |
