# Supplementary material for: Genome-wide characterization and expression analysis of aquaporins in salt cress (Eutrema salsugineum)
Source: PeerJ. 2019 Sep 12;7:e7664. doi: 10.7717/peerj.7664 (PMC6745184; doi:10.7717/peerj.7664)
Supplement: Table S3 — Mismatched names highlighted in Yellow. [file peerj-07-7664-s003.docx]

**Table S3** Compared the EsAQPs in this study to existing annotation at Phytozme. Mismatched names highlighted in Yellow.

| AQP in this study | Phytozome ID | Annotation of EsAQP at Phytozyme |
| --- | --- | --- |
| EsPIP1;1 | Thhalv10006141m | PIP1;1 |
| EsPIP1;2 | Thhalv10001573m | PIP1;2 |
| EsPIP1;3 | Thhalv10008397m | PIP1;4 |
| EsPIP1;4 | Thhalv10028860m | PIP1;4 |
| EsPIP1;5 | Thhalv10025910m | PIP1;4 |
| EsPIP2;1 | Thhalv10010598m | PIP2;1 |
| EsPIP2;2 | Thhalv10017041m | PIP2;2 |
| EsPIP2;3 | Thhalv10017874m | PIP2;2 |
| EsPIP2;4 | Thhalv10014299m | PIP2;4 |
| EsPIP2;5 | Thhalv10010603m | PIP2;5 |
| EsPIP2;6 | Thhalv10017026m | PIP2;6 |
| EsPIP2;7 | Thhalv10025940m | PIP2;7 |
| EsTIP1;1 | Thhalv10017134m | TIP1;1 |
| EsTIP1;2 | Thhalv10004806m | TIP1;2 |
| EsTIP1;3 | Thhalv10028907m | TIP1;3 |
| EsTIP2;1 | Thhalv10021303m | TIP2;1 |
| EsTIP2;2 | Thhalv10026069m | TIP2;2 |
| EsTIP2;3 | Thhalv10001007m | TIP2;3 |
| EsTIP2;4 | Thhalv10011730m | TIP2;1 |
| EsTIP3;1 | Thhalv10019492m | TIP3;1 |
| EsTIP3;2 | Thhalv10008480m | BETA-TIP |
| EsTIP4;1 | Thhalv10002084m | TIP4;1 |
| EsTIP5;1 | Thhalv10011032m | TIP5;1 |
| EsNIP1;2 | Thhalv10025855m | NIP1;2 |
| EsNIP2;1 | Thhalv10017039m | NIP2;1 |
| EsNIP3;1 | Thhalv10008205m | NIP3;1 |
| EsNIP4;1 | Thhalv10027876m | NIP4;1 |
| EsNIP4;2 | Thhalv10028080m | NIP4;1 |
| EsNIP4;3 | Thhalv10028303m | NIP4;1 |
| EsNIP5;1 | Thhalv10028842m | NIP5;1 |
| EsNIP6;1 | Thhalv10018924m | NIP6;1 |
| EsNIP7;1 | Thhalv10021311m | NIP7;1 |
| EsSIP1;1 | Thhalv10021302m | SIP1;1 |
| EsSIP1;2 | Thhalv10014528m | SIP1;2 |
| EsSIP2;1 | Thhalv10006214m | SIP2;1 |
