# Supplementary material for: Genome-wide characterization and expression analysis of aquaporins in salt cress (Eutrema salsugineum)
Source: PeerJ. 2019 Sep 12;7:e7664. doi: 10.7717/peerj.7664 (PMC6745184; doi:10.7717/peerj.7664)
Supplement: Table S4 [file peerj-07-7664-s004.docx]

**Table S4** Predictions of subcellular localization of *EsAQP* genes in WoLF PSORT.

| Name | WoLF PSORT |
| --- | --- |
| EsPIP1;1 | plas:**7^＊^**, cysk:5, cyto:1 |
| EsPIP1;2 | plas:**8**, cysk:2.5, cyto:2, cysk_nucl:2 |
| EsPIP1;3 | plas:**8**, cyto:3, cysk:2 |
| EsPIP1;4 | plas:**7**, cysk:4, cyto:2 |
| EsPIP1;5 | plas:**7**, cysk:6 |
| EsPIP2;1 | plas:**10**, golg:2, vacu:1 |
| EsPIP2;2 | plas:**10**, golg:2, vacu:1 |
| EsPIP2;3 | plas:**10**, golg:2, vacu:1 |
| EsPIP2;4 | plas:**12**, golg:2 |
| EsPIP2;5 | plas:**8.5**, cyto_plas:5.5, pero:2, cyto:1.5, nucl:1 |
| EsPIP2;6 | plas:**10**, golg:2, vacu:1 |
| EsPIP2;7 | plas:**6**, cyto:4, cysk:2.5, cysk_nucl:2 |
| EsTIP1;1 | cyto:**8**, vacu:3, chlo:1, plas:1 |
| EsTIP1;2 | plas:**6**, vacu:**6**, cyto:1 |
| EsTIP1;3 | cyto:**7**, vacu:5, plas:2 |
| EsTIP2;1 | chlo:**5**, vacu:**5**, cyto:1, mito:1, plas:1 |
| EsTIP2;2 | vacu:**11**, golg:2 |
| EsTIP2;3 | vacu:**14** |
| EsTIP2;4 | vacu:**13** |
| EsTIP3;1 | chlo:**3**, cyto:**3**, vacu:**3**, mito:2, plas:2 |
| EsTIP3;2 | chlo:**3**, mito:**3**, vacu:**3**, cyto:2, plas:2 |
| EsTIP4;1 | vacu:**7**, plas:3, cyto:2, nucl:1 |
| EsTIP5;1 | chlo:**11**, mito:3 |
| EsNIP1;2 | plas:**8**, vacu:3, cysk:3 |
| EsNIP2;1 | vacu:**9**, plas:3 |
| EsNIP3;1 | vacu:**7**, golg:3, plas:2, |
| EsNIP4;1 | plas:**6**, nucl:3, cyto:3, E.R.:1 |
| EsNIP4;2 | plas:**5**, cyto:3, pero:2, chlo:1, nucl:1, vacu:1 |
| EsNIP4;3 | plas:**8**, vacu:2, cysk:2, chlo:1 |
| EsNIP5;1 | plas:**8**, vacu:4 |
| EsNIP6;1 | plas:**13** |
| EsNIP7;1 | cyto:**7**, plas:5, mito:1 |
| EsSIP1;1 | plas:**9**, golg:3 |
| EsSIP1;2 | vacu:**6**, plas:4, chlo:2 |
| EsSIP2;1 | E.R:**5**, plas:3.5, vacu:3, |

*Abbreviation: plas, plasma membrane; cyto, cytosol; cysk, cytoskeleton; vacu, tonoplast membrane; chlo, chloroplast; mito, mitochondria; golg,* *golgi apparatus; E.R, endoplasmic reticulum; pero, peroxisome; nucl, nuclear*

***^＊^****represent the value of numbers of nearest neighbors, the maximum value is shown in bold and selected in Table 1.*
