# Supplementary figures and images for: Genome-wide characterization and expression analysis of aquaporins in salt cress (Eutrema salsugineum)
Source: PeerJ. 2019 Sep 12;7:e7664. doi: 10.7717/peerj.7664 (PMC6745184; doi:10.7717/peerj.7664)

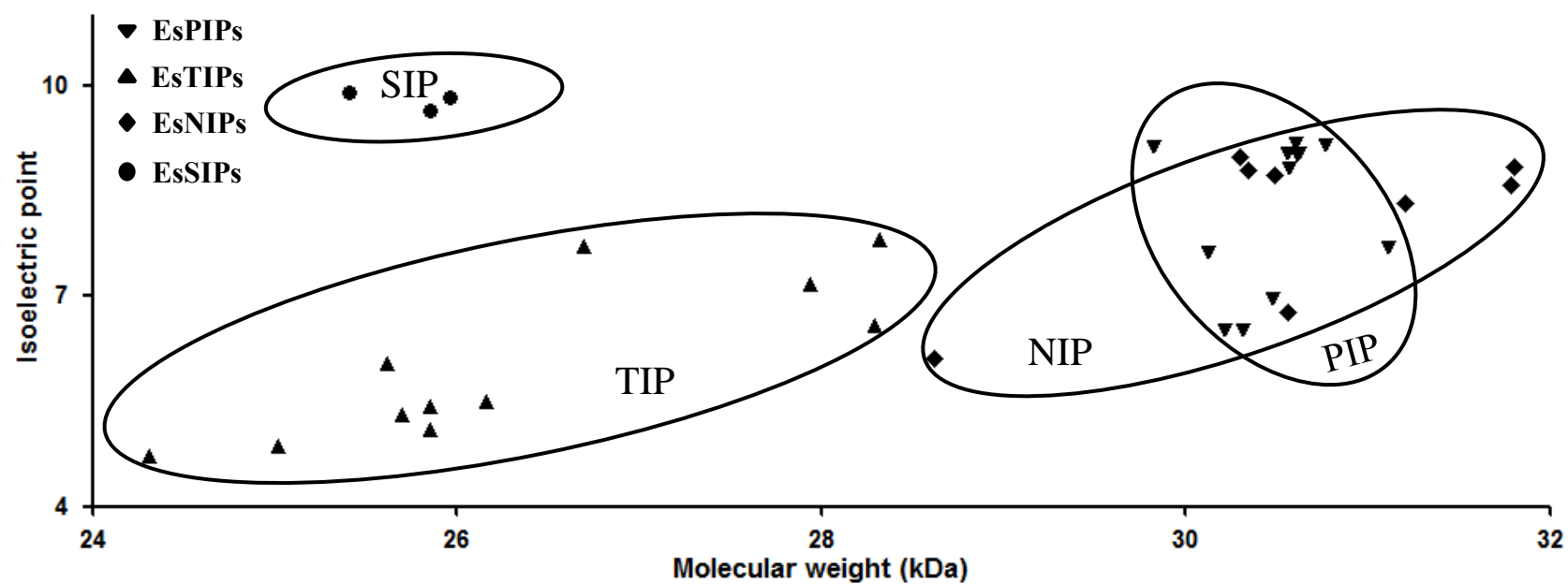

**Figure S1** Putative pI and MW of PIPs, TIPs, NIPs, and SIPs from *E. salsugineum*.

Supplement: Figure S1 [file peerj-07-7664-s005.pdf]
