# Supplementary material for: Genome-wide characterization and expression analysis of aquaporins in salt cress (Eutrema salsugineum)
Source: PeerJ. 2019 Sep 12;7:e7664. doi: 10.7717/peerj.7664 (PMC6745184; doi:10.7717/peerj.7664)
Supplement: Figure S2 — Multiple alignments were performed using ClustalX. The SDPs are highlighted in yellow, mismatch site highlighted in red and the representative sequences are marked in blue. The Genbank accession numbers: AtPIP1;2 (Q06611), AtPIP2;1 (P43286), AtPIP2;4 (Q9FF53), AtTIP1;1 (P25818), AtTIP1;2 (Q41963), AtTIP1;3 (NP_192056), AtTIP2;1 (Q41951), AtTIP2;3 (Q9FGL2), AtTIP4;1 (O82316), AtTIP5;1 (NP_190328), AtNIP1;2 (Q8LFP7), AtNIP5;1 (NP_192776), AtNIP6;1 (NP_178191), CpNIP1 (CAD67694), GmNOD26 (P08995), HvPIP1;3 (BAA23745), HvPIP1;4 (BAF33068), HvPIP2;1 (BAA23744), NtAQP1 (O24662), NtTIPa (Q9XG70), OsNIP2;1 (Q6Z2T3), TaTIP2;1 (AAS19468), TaTIP2;2 (AAS19469), ZmPIP1;1 (Q41870), ZmPIP1;5 (Q9AR14). [file peerj-07-7664-s006.pdf]

Figure S2 Specificity determining positions (SDPs) analysis of *E.salsugineum* AQPs from alignments with putative amino acid sequences of AQPs transporting non-aqua substrates. Multiple alignments were performed using ClustalX. The SDPs are highlighted in yellow, mismatch site highlighted in red and the representative sequences are marked in blue. The Genbank accession numbers: AtPIP1;2 (Q06611), AtPIP2;1 (P43286), AtPIP2;4 (Q9FF53), AtTIP1;1 (P25818), AtTIP1;2 (Q41963), AtTIP1;3 (NP_192056), AtTIP2;1 (Q41951), AtTIP2;3 (Q9FGL2), AtTIP4;1 (O82316), AtTIP5;1 (NP_190328), AtNIP1;2 (Q8LFP7), AtNIP5;1 (NP_192776), AtNIP6;1 (NP_178191), CpNIP1 (CAD67694), GmNOD26 (P08995), HvPIP1;3 (BAA23745), HvPIP1;4 (BAF33068), HvPIP2;1 (BAA23744), NtAQP1 (O24662), NtTIPa (Q9XG70), OsNIP2;1 (Q6Z2T3), TaTIP2;1 (AAS19468), TaTIP2;2 (AAS19469), ZmPIP1;1 (Q41870), ZmPIP1;5 (Q9AR14).

Ammonia Transporters

```
TaTIP2;1     GHVNPAVTFGLALGGQITILTGIFYWVAQLLGAIVGAFLVQFCT--------GVATPTHG
TaTIP2;2     GHVNPAVTFGLALGGQITILTGIFYWVAQLLGAIVGAFLVQFCT--------GVATPTHG
AtTIP2;1     GHVNPAVTFGLAVGGQITVITGVFYWIAQLLGSTAACFLLKYVTG-------GLAVPTHS
AtTIP2;3     GHLNPAVTLGLAIGGNITLITGFFYWIAQCLGSIVACLLLVFVTN-------GKSVPTHG
EsTIP2;1     GHVNPAVTFGLALGGQITLITGVFYWIAQLLGSTAACFLLKFVTG-------GLAVPTHS
EsTIP3;1     GHVNPAVTFGALIGGRLSAIRAIYYWIAQLLGAILACLLLRLSTN-------GLRPVGFR
GmNOD26      GHFNPAVTIAFASTRRFPLIQVPAYVVAQLLGSILASGTLRLLF-------MGNHDQFSG
EsNIP1;2     AHFNPAVTIAFASCRRFPLKQVPAYVISQLIGSTLAAATLRLLFGLDNDVCSGKHDVFVG
EsNIP4;1     AHFNPAVTVTFAIFRRFPWYQVPLYIGAQLTGSLLASLTLRLMF-------KVTPEAFFG
EsNIP4;3     AHFNPAVTITFAIFRRFPWYQVPSYIGAQLAGSLLASLTLRLMF-------KVTPEAFFG
```

```
TaTIP2;1     LS-GVGAFEGVVMEIIVTFGLVYTVYATAADPKKGSLGTIAPIAIGFIVGANILVAGPFS
TaTIP2;2     LS-GVGAFEGVVMEIIVTFGLVYTVYATAADPKKGSLGTIAPIAIGFIVGANILVAGPFS
AtTIP2;1     VAAGLGSIEGVVMEIIITFALVYTVYATAADPKKGSLGTIAPLAIGLIVGANILAAGPFS
AtTIP2;3     VSAGLGAVEGVVMEIVVTFALVYTVYATAADPKKGSLGTIAPIAIGFIVGANILAAGPFS
EsTIP2;1     VAAGVGAIEGVVMEIIITFALVYTVYATAADPKNGSLGTIAPLAIGLIVGANILAAGPFS
EsTIP3;1     LASGVGAVNGLVLEIILTFGLVYVVYSTLIDPKRGSLGIIAPLAIGLIVGANTLVGGPFS
GmNOD26      TVPNGTNLQAFVFEFIMTFFLMFVICGVATDNR--AVGEFAGIAIGSTLLLNVIIGGPVT
EsNIP1;2     TLPSGSDLQSFVIEFIITFYLMFIISGVATDNR--AIGELAGIAVGSTVLLNVIIAGPVS
EsNIP4;1     TTPADSSARALVAEIIISFLLMFVISGVATDNR--AVGELAGIAVGMTIMLNVFVAGPIS
EsNIP4;3     TTPADSSARALVAEIIISFLLMFVISGVATDSR--AIGELAGIAVGMTIMLNVFVAGPIS
```

```
TaTIP2;1     GGSMNPARSFGPAVASGDFTNIWVYWAGPLIGGGLAGVVYRYLYMCD------DHSSVAG
TaTIP2;2     GGSMNPARSFGPAVASGDFTNIWIYWAGPLIGGGLAGVVYRYVYMCD------DHSSVAG
AtTIP2;1     GGSMNPARSFGPAVAAGDFSGHWVYWVGPLIGGGLAGLIYGNVFMGSS-----EHVPLAS
AtTIP2;3     GGSMNPARSFGPAVVSGDLSQIWIYWVGPLVGGALAGLIYGDVFIGSY-----EAVETRE
EsTIP2;1     GGSMNPARSFGPAVAAGDFSGHWVYWVGPLIGGGLAGLIYGNVFMTSS-----EHQPLAS
EsTIP3;1     GASMNPARAFGPALVGWRWHDHWIYWVGPFIGGALAALIYEYMVIPSEPPTHHTHQPLAP
GmNOD26      GASMNPARSLGPAFVHGEYEGIWIYLLAPVVGAIAGAWVYNIVRYTDKPLSETTKSASFL
EsNIP1;2     GASMNPGRSLGPAMVYNCYKGIWIYIVSPILGAIAGAWVYNTVRYTDKPLREITKSGSFL
```

```
EsNIP4;1     GASMNPARSLGPAIVMGVYKGIWIYIVGPLVGIMAGGFVYNLIRFTDKPLRELTRSGSFL
EsNIP4;3     GASMNPARSLGPAIVMGKYKGIWVYIIGPVIGIMAGGFVYNFIRFTDKPLRELTKSASFL
```

Boric Acid Transporters

```
HvPIP1;3     PSGSK----CGTVGIQGIAWSFGGMIFVLVYCTAGISGGHINPAVTFGLFLARKLSLTRA
HvPIP1;4     PSGSK----CGTVGIQGIAWSFGGMIFVLVYCTAGISGGHINPAVTFGLFLARKLSLTRA
ZmPIP1;1     KSTSK----CATVGIQGIAWSFGGMILALVYCTAGISG-HINPAVTFGLFLARKLSLTRA
AtNIP5;1     --------YDGAETLIGNAACAGLAVMIIILSTGHISGAHLNPSLTIAFAALRHFPWAHV
AtNIP6;1     --------TDGAETLIGCAASAGLAVMIVILSTGHISGAHLNPAVTIAFAALKHFPWKHV
OsNIP2;1     --------DLSRISQLGQSIAGGLIVTVMIYAVGHISGAHMNPAVTLAFAVFRHFPWIQV
EsPIP1;1     RSPNM----CSSVGIQGIAWAFGGMIFALVYCTAGISGGHINPAVTFGLFLARKLSFTRA
EsPIP1;2     RSPNM----CASVGIQGIAWAFGGMIFALVYCTAGISGGHINPAVTFGLFLARKLSLTRA
EsPIP1;3     RAPNM----CASVGIQGIAWAFGGMIFALVYCTAGISGGHINPAVTFGLLLARKLSLSRA
EsPIP1;4     RAPNM----CASVGIQGIAWAFGGMIFALVYCTAGISGGHINPAVTFGLFLARKLSLTRA
EsPIP1;5     RAPNM----CASVGIQGIAWAFGGMIFALVYCTAGISGGHINPAVTFGLFLARKLSLTRA
EsPIP2;5     TDPTLNPDQCAGVGILGIAWAFGGMIFILVYCTAGISGGHINPAVTFGLFLARKVTLVRA
EsNIP5;1     --------YDGAETLIGNAACAGLAVMIIILSTGHISGAHLNPSLTIAFAALRHFPWSHV
EsNIP6;1     --------TGGAVTLIGCAASAGLAVMIVILSTGHISGAHLNPAVTIAFAALKHFPWKHV
EsNIP7;1     --------SGGHVGLLEYAATAGLSVVVVVYSVGHISGAHLNPSITIAFAVFGGFPWSQV

HvPIP1;3     VFYIVMQCLGAICGAGVVKGFQTTLYQ-----GNGGGANSVAAGYTKGDGLGAEIVGTFV
HvPIP1;4     VFYIVMQCLGAICGAGVVKGFQTTLYQ-----GNGGGANSVAAGYTKGDGLGAEIVGTFV
ZmPIP1;1     VFYIIMQCLGAICGRGVVKGFQQGLYM-----GNGGRRNVVAPGYTKGDGLGAEIVGTFI
AtNIP5;1     PAYIAAQVSASICASFALKGVFHPF----------MSGGVTIPSVSLGQAFALEFIITFI
AtNIP6;1     PVYIGAQVMASVSAAFALKAVFEPT----------MSGGVTVPTVGLSQAFALEFIISFN
OsNIP2;1     PFYWAAQFTGAICASFVLKAVIHPVD----------VIGTTTPVGPHWHSLVVEVIVTFN
EsPIP1;1     LYYIVMQCLGAICGAGVVKGFQPKQYQ-----SLGGGANTVAHGYTKGSGLGAEIIGTFV
EsPIP1;2     VYYIVMQCLGAICGAGVVKGFQPKQYQ-----ALGGGANTIAHGYTKGSGLGAEIIGTFV
EsPIP1;3     VFYMVMQCLGAICGAGVVKGFQPNPYQ-----TLGGGANTVAHGYTKGSGLGAEIIGTFV
EsPIP1;4     VFYMIMQCLGAICGAGVVKGFQPTPYQ-----TLGGGANTVAPGYTKGSGLGAEIIGTFV
EsPIP1;5     VFYIVMQCLGAICGAGVVKGFQSGPYQ-----ANGGGANVVAHGYTKGSGLGAEIVGTFV
EsPIP2;5     VMYMVAQCLGAICGVALVKSFQSAYYT-----RYGGGANGLADGYSVGTGVAAEIVGTFV
EsNIP5;1     PAYIAAQVSASICASFALKAVFHPF----------MSGGVTVPSSSIGQAFALEFIISFI
EsNIP6;1     PVYIGAQIMASVCAAFALKAVFEPT----------MSGGVTVPTVALSQAFALEFIITFN
EsNIP7;1     PLYITAQTLGATAATLVGVSVYGVN----------ADLMATKPTLSCVSAFCVELVATGI

HvPIP1;3     LVYTVFSATDAKRSARDSHVPILAPLPIGFAVFLVHLATIPITGTGINPARSLGAAIIYN
HvPIP1;4     LVYTVFSATDAKRSARDSHVPILAPLPIGFAVFLVHLATIPITGTGINPARSLGAAIIYN
ZmPIP1;1     LVYTVFSATDAKRRARDSHVPILAPLPIGFAVFLVHLATMGITGTGINPARSLGAAVIYN
AtNIP5;1     LLFVVTAVAT-----DTRAVGELAGIAVGATVMLNILVAGPSTGGSMNPVRTLGPAVASG
AtNIP6;1     LMFVVTAVAT-----DTRAVGELAGIAVGATVMLNILIAGPATSASMNPVRTLGPAIAAN
OsNIP2;1     MMFVTLAVAT-----DTRAVGELAGLAVGSAVCITSIFAGAISGGSMNPARTLGPALASN
EsPIP1;1     LVYTVFSATDAKRNARDSHVPILAPLPIGFAVFLVHLATIPITGTGINPARSLGAAIIYN
```

EsPIP1;2	LVYTVFSATDAKRNARDSHVPILAPLPIGFAVFLVHLATIPITGTGINPARSLGAAIIFN
EsPIP1;3	LVYTVFSATDAKRSARDSHVPILAPLPIGFAVFLVHLATIPITGTGINPARSLGAAIIYN
EsPIP1;4	LVYTVFSATDAKRSARDSHVPILAPLPIGFAVFLVHLATIPITGTGINPARSLGAAIIYN
EsPIP1;5	LVYTVFSATDAKRSARDSHVPILAPLPIGFAVFLVHLATIPITGTGINPARSLGAAIIYN
EsPIP2;5	LVYTVFSATDPKRSARDSHVPVLAPLPIGFAVFIVHLATIPITGTGINPARSLGAAIIYN
EsNIP5;1	LLFVVTAVAT-----DTRAVGELAGIAVGATVMLNILVAGPSSGGSMNPVRTLGPAVASG
EsNIP6;1	LMFVVTAVAT-----DTRAVGELAGIAVGATVMLNILIAGPATSASMNPVRTLGPAIAAN
EsNIP7;1	VVFLASALHCG----PHQNSGNLTGLVIGTVISLGVLITGPISGGSMNPARSLGPAVVAW

CO_2 Transporters

AtPIP1;2	GHINPAVTFGLFLARKLSLTRAVYYIVMQCLGAICGAGVVKGFQPKQYQALGGGANTIAH
NtAQP1	GHINPAVTFGLFLARKLSLTRAIFYIVMQCLGAICGAGVVKGFMVGPYQRLGGGANVVNH
HvPIP2;1	GHINPAVTFGLFLARKVSLIRALLYIIAQCLGAICGVGLVKGFQSSYYVRYGGGANELSA
EsPIP1;1	GHINPAVTFGLFLARKLSFTRALYYIVMQCLGAICGAGVVKGFQPKQYQSLGGGANTVAH
EsPIP1;2	GHINPAVTFGLFLARKLSLTRAVYYIVMQCLGAICGAGVVKGFQPKQYQALGGGANTIAH
EsPIP1;3	GHINPAVTFGLLLARKLSLSRAVFYMVMQCLGAICGAGVVKGFQPNPYQTLGGGANTVAH
EsPIP1;4	GHINPAVTFGLFLARKLSLTRAVFYMIMQCLGAICGAGVVKGFQPTPYQTLGGGANTVAP
EsPIP1;5	GHINPAVTFGLFLARKLSLTRAVFYIVMQCLGAICGAGVVKGFQSGPYQANGGGANVVAH
EsPIP2;4	GHINPAVTFGLFLARKVSLVRTVLYIVAQCLGAICGCGFVKAFQSSYYTRYGGGANELAD

AtPIP1;2	GYTKGSGLGAEIIGTFVLVYTVFSATDAKRNARDSHVPILAPLPIGFAVFLVHLATIPIT
NtAQP1	GYTKGDGLGAEIIGTFVLVYTVFSATDAKRNARDSYVPILAPLPIGFAVFLVHLATIPIT
HvPIP2;1	GYSKGTGLAAEIIGTFVLVYTVFSATDPKRNARDSHIPVLAPLPIGFAVFMVHLATIPIT
EsPIP1;1	GYTKGSGLGAEIIGTFVLVYTVFSATDAKRNARDSHVPILAPLPIGFAVFLVHLATIPIT
EsPIP1;2	GYTKGSGLGAEIIGTFVLVYTVFSATDAKRNARDSHVPILAPLPIGFAVFLVHLATIPIT
EsPIP1;3	GYTKGSGLGAEIIGTFVLVYTVFSATDAKRSARDSHVPILAPLPIGFAVFLVHLATIPIT
EsPIP1;4	GYTKGSGLGAEIIGTFVLVYTVFSATDAKRSARDSHVPILAPLPIGFAVFLVHLATIPIT
EsPIP1;5	GYTKGSGLGAEIVGTFVLVYTVFSATDAKRSARDSHVPILAPLPIGFAVFLVHLATIPIT
EsPIP2;4	GYNKGTGLGAEIIGTFVLVYTVFSATDPKRSARDSHIPVLAPLPIGFAVFMVHLATIPIT

AtPIP1;2	GTGINPARSLGAAIIFNKDNAWDDHWVFWVGPFIGAALAALYHVIVIRAIPFK----SRS
NtAQP1	GTGINPARSLGAAIIYNTDQAWDDHWIFWVGPFIGAALAAVYHQIIIRAIPFH----KSS
HvPIP2;1	GTGINPARSLGAAVIYNTDKAWDDQWIFWVGPLIGAAIAAAYHQYVLRASAAK-LGSYRS
EsPIP1;1	GTGINPARSLGAAIIYNKDHSWDDHWVFWVGPFIGAALAALYHVIVIRAIPFK----SRS
EsPIP1;2	GTGINPARSLGAAIIFNKDNAWDDHWVFWVGPFIGAALAALYHVIVIRAIPFK----SRS
EsPIP1;3	GTGINPARSLGAAIIYNKDHAWDDHWIFWVGPFIGAALAALYHQLVIRAIPFK----TRS
EsPIP1;4	GTGINPARSLGAAIIYNKDHSWDDHWIFWVGPFIGAALAALYHQIVIRAIPFK----SKS
EsPIP1;5	GTGINPARSLGAAIIYNKDHAWDDHWIFWVGPFIGAALAALYHQIVIRAIPFK----SKR
EsPIP2;4	GTGINPARSFGAAVIYNQEKAWDDQWIFWVGPMIGAAAAAALYHQFVLRAAAIKALGSFRS

H_2O_2 Transporters

AtTIP1;1	AVAHAFGLFVAVSVGANISGGHVNPAVTFGAFIGGNITLLRGILYWIAQLLGSVVACLIL

```
AtTIP1;2     ALAHAFGLFVAVSVGANISGGHVNPAVTFGVLLGGNITLLRGILYWIAQLLGSVAACFLL
AtTIP2;3     AIAHAFALFVGVSIAANISGGHLNPAVTLGLAIGGNITLITGFFYWIAQCLGSIVACLLL
AtPIP2;1     AWAFGGMIFILVYCTAGISGGHINPAVTFGLFLARKVSLPRALLYIIAQCLGAICGVGFV
AtPIP2;4     AWAFGGMIFVLVYCTAGISGGHINPAVTVGLFLARKVSLVRTVLYIVAQCLGAICGCGFV
AtNIP1;2     AIVWGLTVMVLVYSLGHISGAHFNPAVTIAFASCGRFPLKQVPAYVISQVIGSTLAAATL
EsPIP1;1     AWAFGGMIFALVYCTAGISGGHINPAVTFGLFLARKLSFTRALYYIVMQCLGAICGAGVV
EsPIP1;2     AWAFGGMIFALVYCTAGISGGHINPAVTFGLFLARKLSLTRAVYYIVMQCLGAICGAGVV
EsPIP1;3     AWAFGGMIFALVYCTAGISGGHINPAVTFGLLLARKLSLSRAVFYMVMQCLGAICGAGVV
EsPIP1;4     AWAFGGMIFALVYCTAGISGGHINPAVTFGLFLARKLSLTRAVFYMIMQCLGAICGAGVV
EsPIP1;5     AWAFGGMIFALVYCTAGISGGHINPAVTFGLFLARKLSLTRAVFYIVMQCLGAICGAGVV
EsPIP2;1     AWAFGGMIFILVYCTAGISGGHINPAVTFGLLLARKVSLVRAILYMVAQCLGAICGVGFV
EsPIP2;2     AWAFGGMIFILVYCTAGISGGHINPAVTFGLFLARKVSLIRAVLYMVAQCLGAICGVGFV
EsPIP2;3     AWAFGGMIFILVYCTAGISGGHINPAVTFGLFLARKVSLIRAVLYMVAQCLGAICGVGFV
EsPIP2;4     AWAFGGMIFVLVYCTAGVSGGHINPAVTFGLFLARKVSLVRTVLYIVAQCLGAICGCGFV
EsPIP2;5     AWAFGGMIFILVYCTAGISGGHINPAVTFGLFLARKVTLVRAVMYMVAQCLGAICGVALV
EsPIP2;6     SWAFGGMIFILVYCTAGISGGHINPAVTFGLFLASKVSLVRAISYMVAQCLGATCGVGLV
EsPIP2;7     AWAFGGMIFVLVYCTAGISGGHINPAVTFGLFLARKVSLVRAVGYMIAQCLGAICGVGFV
EsTIP1;1     ALAHAFGLFVAVSVGANISGGHVNPAVTFGAFVGGNITLLRGILYWIAQLAGSVVACLLL
EsTIP1;2     ALAHAFGLFVAVSVGANISGGHVNPAVTFGAFLGGNITLLRGILYWIAQLLGSVVACFLL
EsTIP1;3     SLSHAFALFVAVSVGANVSGGHVNPAVTFGAFIGGNITLMRAILYWIAQLLGAVVACLLL
EsTIP2;1     AVCHGFALFVAVAIGANISGGHVNPAVTFGLALGGQITLITGVFYWIAQLLGSTAACFLL
EsTIP2;2     AVAHAFALFVGVSIAANISGGHLNPAVTLGLAVGGNITIITGFFYWIAQCLGSIVACLLL
EsTIP2;3     ALAHAFALFVGVSIAANISGGHLNPAVTLGLAVGGNITLITGFLYWIAQCLGSIVACLLL
EsTIP3;2     ALAHALALFAAVSAAINVSGGHVNPAVTLGALFGGRISVIRAVYYWIAQLLGAILACLLL
EsTIP4;1     AVAHAFVVAVMISAG-HISGGHLNPAVTIGLLFGGHITVFRAFLYWIDQLLASSAACFLL
EsNIP1;2     AAVWGLTVMVLVYSLGHISGAHFNPAVTIAFASCRRFPLKQVPAYVISQLIGSTLAAATL
EsNIP3;1     ALVWGLTVTVMIYSIGHVSGAHFNPAVSIAFASSKKFPFKQVPAYIAAQVLGSTLAAAAL
EsNIP5;1     AACAGLAVMIIILSTGHISGAHLNPSLTIAFAALRHFPWSHVPAYIAAQVSASICASFAL

AtTIP1;1     KFATGG----LAVPAFGLSAGVGVLN---AFVFEIVMTFGLVYTVYATAID---PKNGSL
AtTIP1;2     SFATGG----EPIPAFGLSAGVGSLN---ALVFEIVMTFGLVYTVYATAVD---PKNGSL
AtTIP2;3     VFVTNG----KSVPTHGVSAGLGAVE---GVVMEIVVTFALVYTVYATAAD---PKKGSL
AtPIP2;1     KAFQSSYYTRYGGGANSLADGYSTGT---GLAAEIIGTFVLVYTVFSATDPKRSARDSHV
AtPIP2;4     KAFQSSYYTRYGGGANELADGYNKGT---GLGAEIIGTFVLVYTVFSATDPKRNARDSHV
AtNIP1;2     RLLFGLDQDVCSGKHDVFVGTLPSGSNLQSFVIEFIITFYLMFVISGVATD-----NRAI
EsPIP1;1     KGFQPKQYQSLGGGANTVAHGYTKGS---GLGAEIIGTFVLVYTVFSATDAKRNARDSHV
EsPIP1;2     KGFQPKQYQALGGGANTIAHGYTKGS---GLGAEIIGTFVLVYTVFSATDAKRNARDSHV
EsPIP1;3     KGFQPNPYQTLGGGANTVAHGYTKGS---GLGAEIIGTFVLVYTVFSATDAKRSARDSHV
EsPIP1;4     KGFQPTPYQTLGGGANTVAPGYTKGS---GLGAEIIGTFVLVYTVFSATDAKRSARDSHV
EsPIP1;5     KGFQSGPYQANGGGANVVAHGYTKGS---GLGAEIVGTFVLVYTVFSATDAKRSARDSHV
EsPIP2;1     KAFQSSYYIRYGGGANSLAEGYSTGT---GLAAEIIGTFVLVYTVFSATDPKRSARDSHV
EsPIP2;2     KAFQSSYYVRYGGGANSLAEGYSTGT---GLAAEIIGTFVLVYTVFSATDPKRNARDSHV
EsPIP2;3     KAFQSSYYVRYGGGANSLADGYNTGT---GLAAEIIGTFVLVYTVFSATDPKRNARDSHV
EsPIP2;4     KAFQSSYYTRYGGGANELADGYNKGT---GLGAEIIGTFVLVYTVFSATDPKRSARDSHI
```

```
EsPIP2;5    KSFQSAYYTRYGGGANGLADGYSVGT---GVAAEIVGTFVLVYTVFSATDPKRSARDSHV
EsPIP2;6    KIFQSTYYNRYGGGANVLADGYNVGV---GVGAEIIGTFVLVYTVFSATDPKRNARDSHI
EsPIP2;7    KAFMKTPYNTLGGGANTVADGYSKGT---ALGAEIIGTFVLVYTVFSATDPKRSARDSHI
EsTIP1;1    KFATGG----LVVPAFGLSAGVGVLN---AFVFEIVMTFGLVYTVYATAID---PKNGSL
EsTIP1;2    TFATGG----LAVPAFGLSAGVGSLN---AFVFEIVMTFGLVYTVYATAID---PKNGSL
EsTIP1;3    KVSTGG----METAAFSLSYGVTPWN---AVVFEIVMTFGLVYTVYATAVD---PKKGDI
EsTIP2;1    KFVTGG----LAVPTHSVAAGVGAIE---GVVMEIIITFALVYTVYATAAD---PKNGSL
EsTIP2;2    VFVTNG----ESVPTHGVAAGLGAVE---GIVMEIVVTFALVYTVYATAAD---PKKGSL
EsTIP2;3    VFVTNG----ESVPTHGVGAGLGAVE---GIVMEIVVTFALVYTVYATAAD---PKKGSL
EsTIP3;2    RLATNG----SRPIGYHVASGVGELH---GLLMEIILTFALVYVVYSTSID---PKRGSI
EsTIP4;1    SYLTGG----MGTPVHTLASGISYTQ---GIIWEIILTFSLLFTVYATIVD---PKKGSL
EsNIP1;2    RLLFGLDNDVCSGKHDVFVGTLPSGSDLQSFVIEFIITFYLMFIISGVATD-----NRAI
EsNIP3;1    RLVFHLNDNVCSLKGDVYVGTYPSNSNTTSFVMEFITTFNLMFVIAAVATD-----KRAT
EsNIP5;1    KAVFHP---FMSGG-----VTVPSSSIGQAFALEFIISFILLFVVTAVATD-----TRAV

AtTIP1;1    GTIAPIAIGFIVGANILAGGAFSGASMNPAVAFGPAVVSWT---WTNHWVYWAGPLVGGG
AtTIP1;2    GTIAPIAIGFIVGANILAGGAFSGASMNPAVAFGPAVVSWT---WTNHWVYWAGPLIGGG
AtTIP2;3    GTIAPIAIGFIVGANILAAGPFSGGSMNPARSFGPAVVSGD---LSQIWIYWVGPLVGGA
AtPIP2;1    PVLAPLPIGFAVFMVHLATIPITGTGINPARSFGAAVIYNKSKPWDDHWIFWVGPFIGAA
AtPIP2;4    PVLAPLPIGFAVFMVHLATIPITGTGINPARSFGAAVIYNNEKAWDDQWIFWVGPMIGAA
AtNIP1;2    GELAGLAVGSTVLLNVIIAGPVSGASMNPGRSLGPAMVYSC---YRGLWIYIVSPIVGAV
EsPIP1;1    PILAPLPIGFAVFLVHLATIPITGTGINPARSLGAAIIYNKDHSWDDHWVFWVGPFIGAA
EsPIP1;2    PILAPLPIGFAVFLVHLATIPITGTGINPARSLGAAIIFNKDNAWDDHWVFWVGPFIGAA
EsPIP1;3    PILAPLPIGFAVFLVHLATIPITGTGINPARSLGAAIIYNKDHAWDDHWIFWVGPFIGAA
EsPIP1;4    PILAPLPIGFAVFLVHLATIPITGTGINPARSLGAAIIYNKDHSWDDHWIFWVGPFIGAA
EsPIP1;5    PILAPLPIGFAVFLVHLATIPITGTGINPARSLGAAIIYNKDHAWDDHWIFWVGPFIGAA
EsPIP2;1    PVLAPLPIGFAVFMVHLATIPITGTGINPARSFGAAVIFNESKPWDDHWIFWVGPFIGAA
EsPIP2;2    PVLAPLPIGFAVFMVHLATIPITGTGINPARSFGAAVIYNESKPWDDHWIFWVGPFIGAA
EsPIP2;3    PVLAPLPIGFAVFMVHLATIPITGTGINPARSFGAAVIFNESKPWDDHWIFWVGPFIGAA
EsPIP2;4    PVLAPLPIGFAVFMVHLATIPITGTGINPARSFGAAVIYNQEKAWDDQWIFWVGPMIGAA
EsPIP2;5    PVLAPLPIGFAVFIVHLATIPITGTGINPARSLGAAIIYNKDQAWDHHWIFWVGPFAGAA
EsPIP2;6    PVLAPLPIGFSVFMVHLATIPITGTGINPARSFGAAVVYNNQKAWDDQWIFWVGPFVGAA
EsPIP2;7    PVLAPLPIGFAVFMVHLATIPITGTGINPARSFGAAVIYNNKKAWDDHWIFWVGPFLGAL
EsTIP1;1    GTIAPIAIGFIVGANILAGGAFSGASMNPAVAFGPAVVSWS---WSNHWVYWAGPLVGGG
EsTIP1;2    GTIAPIAIGFIVGANILAGGAFSGASMNPAVAFGPAVVSWS---WSNHWVYWAGPLVGGG
EsTIP1;3    GIIAPLAIGLIVGANILVGGAFDGASMNPAVSFGPAVVSWT---WTNHWVYWVGPFIGAA
EsTIP2;1    GTIAPLAIGLIVGANILAAGPFSGGSMNPARSFGPAVAAGD---FSGHWVYWVGPLIGGG
EsTIP2;2    GTIAPIAIGFIVGANILAAGPFSGGSINPARSFGPAVVSGD---FSQIWIYWVGPLVGGA
EsTIP2;3    GTIAPIAIGFIVGANILAAGPFSGGSMNPARSFGPAVVSGD---LSQIWIYWVGPLVGGA
EsTIP3;2    GIIAPLAIGLIFGANMLVGGPFDGASMNPARTFGPALVGWR---WHNHWIYWVGPFIGGA
EsTIP4;1    DGLGPLLTGFVVGANILAGGAFSGASMNPARSFGPALVSGN---WTDHWVYWVGPLIGGG
EsNIP1;2    GELAGIAVGSTVLLNVIIAGPVSGASMNPGRSLGPAMVYNC---YKGIWIYIVSPILGAI
EsNIP3;1    GSFAGVAIGATVVLDILMSGPISGASMNPARSLGPAFIWGC---YKDLWLYIVSPVLGAL
EsNIP5;1    GELAGIAVGATVMLNILVAGPSSGGSMNPVRTLGPAVASGN---YRSLWVYLVAPTLGAI
```

Silicic Acid Transporters (not found)

Urea Transporters

NtAQP1	SSVGIQGVAWAFGGMIFALVYCTAGISGGHINPAVTFGLFLARKLSLTRAIFYIVMQCLG
ZmPIP1;5	ATVGIQGIAWSFGGMIFALVYCTAGISGGHINPAVTFGLFLARKLSLTRALFYMVMQCLG
NtTIPa	PLVSLFFVAMAHALVVAVTISAGFRISGGHLNPAVTLGLCMGGHITVFRSILYWIDQLLA
AtTIP4;1	TLVGLFAVAVAHAFVVAVMISAG-HISGGHLNPAVTLGLLLGGHISVFRAFLYWIDQLLA
AtTIP1;1	TPSGLVAAAVAHAFGLFVAVSVGANISGGHVNPAVTFGAFIGGNITLLRGILYWIAQLLG
AtTIP1;2	TPSGLVAAALAHAFGLFVAVSVGANISGGHVNPAVTFGVLLGGNITLLRGILYWIAQLLG
AtTIP1;3	TPAGLVAASLSHAFALFVAVSVGANVSGGHVNPAVTFGAFIGGNITLLRAILYWIAQLLG
AtTIP2;1	DTPGLVAIAVCHGFALFVAVAIGANISGGHVNPAVTFGLAVGGQITVITGVFYWIAQLLG
AtTIP5;1	GPFGVLIPAIANALALSSSVYISWNVSGGHVNPAVTFAMAVAGRISVPTAMFYWTSQMIA
CpNIP1	--VSQLGASVAGGLIVTVMIYAVGHISGAHMNPAVTTAFAATRHFPWKQVPLYAAAQLSG
OsNIP2;1	--ISQLGQSIAGGLIVTVMIYAVGHISGAHMNPAVTLAFAVFRHFPWIQVPFYWAAQFTG
AtNIP6;1	--ETLIGCAASAGLAVMIVILSTGHISGAHLNPAVTIAFAALKHFPWKHVPVYIGAQVMA
EsPIP1;1	SSVGIQGIAWAFGGMIFALVYCTAGISGGHINPAVTFGLFLARKLSFTRALYYIVMQCLG
EsPIP1;2	ASVGIQGIAWAFGGMIFALVYCTAGISGGHINPAVTFGLFLARKLSLTRAVYYIVMQCLG
EsPIP1;3	ASVGIQGIAWAFGGMIFALVYCTAGISGGHINPAVTFGLLLARKLSLSRAVFYMVMQCLG
EsPIP1;4	ASVGIQGIAWAFGGMIFALVYCTAGISGGHINPAVTFGLFLARKLSLTRAVFYMIMQCLG
EsPIP1;5	ASVGIQGIAWAFGGMIFALVYCTAGISGGHINPAVTFGLFLARKLSLTRAVFYIVMQCLG
EsPIP2;1	GGVGILGIAWAFGGMIFILVYCTAGISGGHINPAVTFGLLLARKVSLVRAILYMVAQCLG
EsPIP2;2	GGVGILGIAWAFGGMIFILVYCTAGISGGHINPAVTFGLFLARKVSLIRAVLYMVAQCLG
EsPIP2;3	GGVGILGIAWAFGGMIFILVYCTAGISGGHINPAVTFGLFLARKVSLIRAVLYMVAQCLG
EsPIP2;4	GGVGILGIAWAFGGMIFVLVYCTAGVSGGHINPAVTFGLFLARKVSLVRTVLYIVAQCLG
EsPIP2;5	AGVGILGIAWAFGGMIFILVYCTAGISGGHINPAVTFGLFLARKVTLVRAVMYMVAQCLG
EsPIP2;6	ASVGLLGISWAFGGMIFILVYCTAGISGGHINPAVTFGLFLASKVSLVRAISYMVAQCLG
EsPIP2;7	DGVGLLGIAWAFGGMIFVLVYCTAGISGGHINPAVTFGLFLARKVSLVRAVGYMIAQCLG
EsTIP1;1	TPSGLVAAALAHAFGLFVAVSVGANISGGHVNPAVTFGAFVGGNITLLRGILYWIAQLAG
EsTIP1;2	TPSGLVAAALAHAFGLFVAVSVGANISGGHVNPAVTFGAFLGGNITLLRGILYWIAQLLG
EsTIP1;3	TPSGLVAASLSHAFALFVAVSVGANVSGGHVNPAVTFGAFIGGNITLMRAILYWIAQLLG
EsTIP2;1	DTPGLVAIAVCHGFALFVAVAIGANISGGHVNPAVTFGLALGGQITLITGVFYWIAQLLG
EsTIP2;2	DPAGLVAVAVAHAFALFVGVSIAANISGGHLNPAVTLGLAVGGNITIITGFFYWIAQCLG
EsTIP2;3	DPAGLVAIALAHAFALFVGVSIAANISGGHLNPAVTLGLAVGGNITLITGFLYWIAQCLG
EsTIP2;4	DASGLVAIAVCHGFALFVAVSIAANHSGGHVNPAVTFGLVLGGKLKIVTGLVYWVAQLLG
EsTIP3;1	TPGGLVLVALAHAFALFAAVSAAINVSGGHVNPAVTFGALIGGRLSAIRAIYYWIAQLLG
EsTIP3;2	TPGGLVLVALAHALALFAAVSAAINVSGGHVNPAVTLGALFGGRISVIRAVYYWIAQLLG
EsTIP4;1	TLVGLFAVAVAHAFVVAVMISAG-HISGGHLNPAVTIGLLFGGHITVFRAFLYWIDQLLA
EsTIP5;1	GPFSVLIPAIANAFALSSSVYISWNVSGGHVNPAVTFGMAIAGRISVPTAMFYWTSQMLA
EsNIP1;2	--VTLPGIAAVWGLTVMVLVYSLGHISGAHFNPAVTIAFASCRRFPLKQVPAYVISQLIG
EsNIP2;1	--VTLVGIAVVWGLVIMVLVYTLGHTS-AHFNPAVTIALASTQRFPLHHVPAYLTVQVIG
EsNIP3;1	--VTLPGIALVWGLTVTVMIYSIGHVSGAHFNPAVSIAFASSKKFPFKQVPAYIAAQVLG
EsNIP4;1	--ITFPGICVTWGLIVMVMIYSTGHISGAHFNPAVTVTFAIFRRFPWYQVPLYIGAQLTG

EsNIP4;2	--VTFPGVCITWGLIVMVMIYSTGHISGAHFNPAVTFTFAIFRRFPWYQVPLYIGAQLVG
EsNIP4;3	--VTFPGICVTWGLIVMVMIYSIGHISGAHFNPAVTITFAIFRRFPWYQVPSYIGAQLAG
EsNIP5;1	--ETLIGNAACAGLAVMIIILSTGHISGAHLNPSLTIAFAALRHFPWSHVPAYIAAQVSA
EsNIP6;1	--VTLIGCAASAGLAVMIVILSTGHISGAHLNPAVTIAFAALKHFPWKHVPVYIGAQIMA
EsNIP7;1	--VGLLEYAATAGLSVVVVVYSVGHISGAHLNPSITIAFAVFGGFPWSQVPLYITAQTLG

NtAQP1	AICGAGVVKGFMVGPYQR---LGGGANVVNHGYTKGDGLGAEIIGTFVLVYTVFSATDAK
ZmPIP1;5	AICGAGVVKGFQEGLYMG---AGGGANAVNPGYTKGDGLGAEIVGTFVLVYTVFSATDAK
NtTIPa	SVAACALLNYLTAG-------LETPVHTLANGVSYGQGIIMEVILTFSLLFTVYTTIVDP
AtTIP4;1	SSAACFLLSYLTGG-------MGTPVHTLASGVSYTQGIIWEIILTFSLLFTVYATIVDP
AtTIP1;1	SVVACLILKFATGG-------LAVPAFGLSAGVGVLNAFVFEIVMTFGLVYTVYATAIDP
AtTIP1;2	SVAACFLLSFATGG-------EPIPAFGLSAGVGSLNALVFEIVMTFGLVYTVYATAVDP
AtTIP1;3	AVVACLLLKVSTGG-------METAAFSLSYGVTPWNAVVFEIVMTFGLVYTVYATAVDP
AtTIP2;1	STAACFLLKYVTGG-------LAVPTHSVAAGLGSIEGVVMEIIITFALVYTVYATAADP
AtTIP5;1	SVMACLVLKVTVME-------QHVPIYKIAGEMTGFGASVLEGVLAFVLVYTVFT-ASDP
CpNIP1	ATCAAFTLRLLLHPIKHLG--------TTTPSGSDLQALVMEIVVTFSMMFVTCAVAT--
OsNIP2;1	AICASFVLKAVIHPVDVIG--------TTTPVGPHWHSLVVEVIVTFNMMFVTLAVAT--
AtNIP6;1	SVSAAFALKAVFEPTMSG--------GVTVPTVGLSQAFALEFIISFNLMFVVTAVAT--
EsPIP1;1	AICGAGVVKGFQPKQYQS---LGGGANTVAHGYTKGSGLGAEIIGTFVLVYTVFSATDAK
EsPIP1;2	AICGAGVVKGFQPKQYQA---LGGGANTIAHGYTKGSGLGAEIIGTFVLVYTVFSATDAK
EsPIP1;3	AICGAGVVKGFQPNPYQT---LGGGANTVAHGYTKGSGLGAEIIGTFVLVYTVFSATDAK
EsPIP1;4	AICGAGVVKGFQPTPYQT---LGGGANTVAPGYTKGSGLGAEIIGTFVLVYTVFSATDAK
EsPIP1;5	AICGAGVVKGFQSGPYQA---NGGGANVVAHGYTKGSGLGAEIVGTFVLVYTVFSATDAK
EsPIP2;1	AICGVGFVKAFQSSYYIR---YGGGANSLAEGYSTGTGLAAEIIGTFVLVYTVFSATDPK
EsPIP2;2	AICGVGFVKAFQSSYYVR---YGGGANSLAEGYSTGTGLAAEIIGTFVLVYTVFSATDPK
EsPIP2;3	AICGVGFVKAFQSSYYVR---YGGGANSLADGYNTGTGLAAEIIGTFVLVYTVFSATDPK
EsPIP2;4	AICGCGFVKAFQSSYYTR---YGGGANELADGYNKGTGLGAEIIGTFVLVYTVFSATDPK
EsPIP2;5	AICGVALVKSFQSAYYTR---YGGGANGLADGYSVGTGVAAEIVGTFVLVYTVFSATDPK
EsPIP2;6	ATCGVGLVKIFQSTYYNR---YGGGANVLADGYNVGVGVGAEIIGTFVLVYTVFSATDPK
EsPIP2;7	AICGVGFVKAFMKTPYNT---LGGGANTVADGYSKGTALGAEIIGTFVLVYTVFSATDPK
EsTIP1;1	SVVACLLLKFATGG-------LVVPAFGLSAGVGVLNAFVFEIVMTFGLVYTVYATAIDP
EsTIP1;2	SVVACFLLTFATGG-------LAVPAFGLSAGVGSLNAFVFEIVMTFGLVYTVYATAIDP
EsTIP1;3	AVVACLLLKVSTGG-------METAAFSLSYGVTPWNAVVFEIVMTFGLVYTVYATAVDP
EsTIP2;1	STAACFLLKFVTGG-------LAVPTHSVAAGVGAIEGVVMEIIITFALVYTVYATAADP
EsTIP2;2	SIVACLLLVFVTNG-------ESVPTHGVAAGLGAVEGIVMEIVVTFALVYTVYATAADP
EsTIP2;3	SIVACLLLVFVTNG-------ESVPTHGVGAGLGAVEGIVMEIVVTFALVYTVYATAADP
EsTIP2;4	STVACFLLKFTTGG-------LAIPIHSVAAGVGSIEGVVMEIIITFALVYTVYATAVDP
EsTIP3;1	AILACLLLRLSTNG-------LRPVGFRLASGVGAVNGLVLEIILTFGLVYVVYSTLIDP
EsTIP3;2	AILACLLLRLATNG-------SRPIGYHVASGVGELHGLLMEIILTFALVYVVYSTSIDP
EsTIP4;1	SSAACFLLSYLTGG-------MGTPVHTLASGISYTQGIIWEIILTFSLLFTVYATIVDP
EsTIP5;1	SVMACLVLKVTVIE-------QHVPIYKIAGEMTGFGASVLEGVLAFVLVYTVFT-ANDP
EsNIP1;2	STLAAATLRLLFGLDNDVCSGKHDVFVGTLPSGSDLQSFVIEFIITFYLMFIISGVAT--
EsNIP2;1	STLASATLRLLFDLNNDVCSKKHDVFLGSSPSGSDLQAFVMEFVITCFLMLVVCAITT—
EsNIP3;1	STLAAAALRLVFHLNDNVCSLKGDVYVGTYPSNSNTTSFVMEFITTFNLMFVIAAVAT--

```
EsNIP4;1    SLLASLTLRLMFKVTP-------EAFFGTTPADSSARALVAEIIISFLLMFVISGVAT—
EsNIP4;2    SLLASLTLRLMFKVTP-------EAYFGTTPIDSAARALVAEIIISFLLMFVISGVAT--
EsNIP4;3    SLLASLTLRLMFKVTP-------EAFFGTTPADSSARALVAEIIISFLLMFVISGVAT--
EsNIP5;1    SICASFALKAVFHPFMSG--------GVTVPSSSIGQAFALEFIISFILLFVVTAVAT--
EsNIP6;1    SVCAAFALKAVFEPTMSG--------GVTVPTVALSQAFALEFIITFNLMFVVTAVAT—
EsNIP7;1    ATAATLVGVSVYGVNADL--------MATKPTLSCVSAFCVELVATGIVVFLASALHCG-

NtAQP1      RNARDSYVPILAPLPIGFAVFLVHLATIPITGTGINPARSLGAAIIYNTDQAWDDHWIFW
ZmPIP1;5    RSARDSHVPILAPLPIGFAVFLVHLATIPITGTGINPARSLGAAIVYNRSHAWNDHWIFW
NtTIPa      K---KGILEGMGPLLTGLVVGANIMAGGPFSGASMNPARSFGPAFVSG---IWTDHWVYW
AtTIP4;1    K---KGSLDGFGPLLTGFVVGANILAGGAFSGASMNPARSFGPALVSG---NWTDHWVYW
AtTIP1;1    K---NGSLGTIAPIAIGFIVGANILAGGAFSGASMNPAVAFGPAVVSW---TWTNHWVYW
AtTIP1;2    K---NGSLGTIAPIAIGFIVGANILAGGAFSGASMNPAVAFGPAVVSW---TWTNHWVYW
AtTIP1;3    K---KGDIGIIAPLAIGLIVGANILVGGAFDGASMNPAVSFGPAVVSW---IWTNHWVYW
AtTIP2;1    K---KGSLGTIAPLAIGLIVGANILAAGPFSGGSMNPARSFGPAVAAG---DFSGHWVYW
AtTIP5;1    R---RGLPLAVGPIFIGFVAGANVLAAGPFSGGSMNPACAFGSAMVYG---SFKNQAVYW
CpNIP1      ---DTKAVGELAGLAVGSAVCITSILAGPVSGGSMNPVRTLGPAMASD---NYKGLWVYF
OsNIP2;1    ---DTRAVGELAGLAVGSAVCITSIFAGAISGGSMNPARTLGPALASN---KFDGLWIYF
AtNIP6;1    ---DTRAVGELAGIAVGATVMLNILIAGPATSASMNPVRTLGPAIAAN---NYRAIWVYL
EsPIP1;1    RNARDSHVPILAPLPIGFAVFLVHLATIPITGTGINPARSLGAAIIYNKDHSWDDHWVFW
EsPIP1;2    RNARDSHVPILAPLPIGFAVFLVHLATIPITGTGINPARSLGAAIIFNKDNAWDDHWVFW
EsPIP1;3    RSARDSHVPILAPLPIGFAVFLVHLATIPITGTGINPARSLGAAIIYNKDHAWDDHWIFW
EsPIP1;4    RSARDSHVPILAPLPIGFAVFLVHLATIPITGTGINPARSLGAAIIYNKDHSWDDHWIFW
EsPIP1;5    RSARDSHVPILAPLPIGFAVFLVHLATIPITGTGINPARSLGAAIIYNKDHAWDDHWIFW
EsPIP2;1    RSARDSHVPVLAPLPIGFAVFMVHLATIPITGTGINPARSFGAAVIFNESKPWDDHWIFW
EsPIP2;2    RNARDSHVPVLAPLPIGFAVFMVHLATIPITGTGINPARSFGAAVIYNESKPWDDHWIFW
EsPIP2;3    RNARDSHVPVLAPLPIGFAVFMVHLATIPITGTGINPARSFGAAVIFNESKPWDDHWIFW
EsPIP2;4    RSARDSHIPVLAPLPIGFAVFMVHLATIPITGTGINPARSFGAAVIYNQEKAWDDQWIFW
EsPIP2;5    RSARDSHVPVLAPLPIGFAVFIVHLATIPITGTGINPARSLGAAIIYNKDQAWDHHWIFW
EsPIP2;6    RNARDSHIPVLAPLPIGFSVFMVHLATIPITGTGINPARSFGAAVVYNNQKAWDDQWIFW
EsPIP2;7    RSARDSHIPVLAPLPIGFAVFMVHLATIPITGTGINPARSFGAAVIYNNKKAWDDHWIFW
EsTIP1;1    K---NGSLGTIAPIAIGFIVGANILAGGAFSGASMNPAVAFGPAVVSW---SWSNHWVYW
EsTIP1;2    K---NGSLGTIAPIAIGFIVGANILAGGAFSGASMNPAVAFGPAVVSW---SWSNHWVYW
EsTIP1;3    K---KGDIGIIAPLAIGLIVGANILVGGAFDGASMNPAVSFGPAVVSW---TWTNHWVYW
EsTIP2;1    K---NGSLGTIAPLAIGLIVGANILAAGPFSGGSMNPARSFGPAVAAG---DFSGHWVYW
EsTIP2;2    K---KGSLGTIAPIAIGFIVGANILAAGPFSGGSINPARSFGPAVVSG---DFSQIWIYW
EsTIP2;3    K---KGSLGTIAPIAIGFIVGANILAAGPFSGGSMNPARSFGPAVVSG---DLSQIWIYW
EsTIP2;4    K---NGTLGTIAPLAIGLIVGANILAAGPFSGGSMNPARSFGPALAAG---DFSGHWVYW
EsTIP3;1    K---RGSLGIIAPLAIGLIVGANTLVGGPFSGASMNPARAFGPALVGW---RWHDHWIYW
EsTIP3;2    K---RGSIGIIAPLAIGLIFGANMLVGGPFDGASMNPARTFGPALVGW---RWHNHWIYW
EsTIP4;1    K---KGSLDGLGPLLTGFVVGANILAGGAFSGASMNPARSFGPALVSG---NWTDHWVYW
EsTIP5;1    R---RGLPLAVGPIFIGFVAGANVLAAGPFSGGSMNPACAFGSAMVYG---SFKNQAVYW
EsNIP1;2    ---DNRAIGELAGIAVGSTVLLNVIIAGPVSGASMNPGRSLGPAMVYN---CYKGIWIYI
EsNIP2;1    ---SKRTSEELEGLIIGATVTLNVIFAGEVSGASMNPARSLGPALVWG---CYKGIWIYL
```

```
EsNIP3;1    ---DKRATGSFAGVAIGATVVLDILMSGPISGASMNPARSLGPAFIWG---CYKDLWLYI
EsNIP4;1    ---DNRAVGELAGIAVGMTIMLNVFVAGPISGASMNPARSLGPAIVMG---VYKGIWIYI
EsNIP4;2    ---DNRAIGELAGIAVGMTIMLNVFVAGPISGASMNPARSLGPAIVMG---VYKNIWVYI
EsNIP4;3    ---DSRAIGELAGIAVGMTIMLNVFVAGPISGASMNPARSLGPAIVMG---KYKGIWVYI
EsNIP5;1    ---DTRAVGELAGIAVGATVMLNILVAGPSSGGSMNPVRTLGPAVASG---NYRSLWVYL
EsNIP6;1    ---DTRAVGELAGIAVGATVMLNILIAGPATSASMNPVRTLGPAIAAN---DYRAIWVYL
EsNIP7;1    ---PHQNSGNLTGLVIGTVISLGVLITGPISGGSMNPARSLGPAVVAW---DFEDIWVYL
```
